# Dorsal and Ventral Dimelia in the Same Hand in A Patient with Severe Ulnar Ray Deficiency: A Case Report

**DOI:** 10.29252/wjps.8.1.112

**Published:** 2019-01

**Authors:** Mohammad Mohammad, F. S. Al-Kahtani

**Affiliations:** Division of Plastic Surgery at King Saud University and King Faisal Specialist Hospital and Research Center, Riyadh, Saudi Arabia

**Keywords:** Dorsal dimelia, Palmar nail, Ventral dimelia, Congenital, Hand

## Abstract

Dorsal dimelia (the appearance of dorsal hand structures on the palmar aspect of the hand) and ventral dimelia (the appearance of ventral hand structures on the dorsal aspect of the hand) are rare congenital anomalies of the hand. None of the previously reported cases had combined dorsal and ventral dimelia in the same patient. Here, we report a case of severe ulnar ray deficiency. The hand had two digits: the radial digit had a palmar nail (dorsal dimelia) and the ulnar digit had absence of the normal dorsal nail along with the appearance of an ectopic pulp on the dorsal aspect of the digit (ventral dimelia). Ulnar ray deficiency is an error of sonic hedgehog (SHH) responsible for antero-posterior patterning of the limb in-utero. Ventral and dorsal dimelia are errors of dorso-ventral patterning of the hand. The complex interactions of SHH with the dorso-ventral axis of development may explain the concurrent dimelia in our patient.

## INTRODUCTION

The appearance of dorsal hand structures on the palmar aspect of the hand is rare and is known as dorsal dimelia. Al-Qattan^1^ subclassified dorsal dimelia into two types. The proximal type is extremely rare and has only been reported in one family.^2^ In this proximal type, the digits and the distal part of the palms are normal. However, the proximal palmar skin is hairy and hyperpigmented; with loss of the normal palmar creases. In the distal type, there is variable dorsalization of the distal palmar skin and digits. The most common manifestation of distal dorsal dimelia is the appearance of a palmar nail; and hence the anomaly has also been named as the congenital palmar nail syndrome,^3^^,^^4^ the double nail deformity,^5^ and the circumferential nail deformity.^6^

The appearance of ventral hand structures on the dorsal aspect of the hand is also rare and is known as ventral dimelia.^1^ In the fully expressed deformity, the dorsal aspect of the entire hand mimics the palmar aspect of the hand; and hence it has also been named as the congenital duplication of the palm deformity.^7^ In the partially expressed deformity, the normal dorsal nail is absent or hypoplastic, along with the appearance of palmar skin on the dorsal aspect of the affected digit.

To our knowledge, the appearance of dorsal and ventral dimelia in the same patient has never been reported. We report a patient with severe ulnar ray deficiency who presented with combined dorsal and ventral dimelia in the same hand. 

## CASE REPORT

**Fig. 1:**
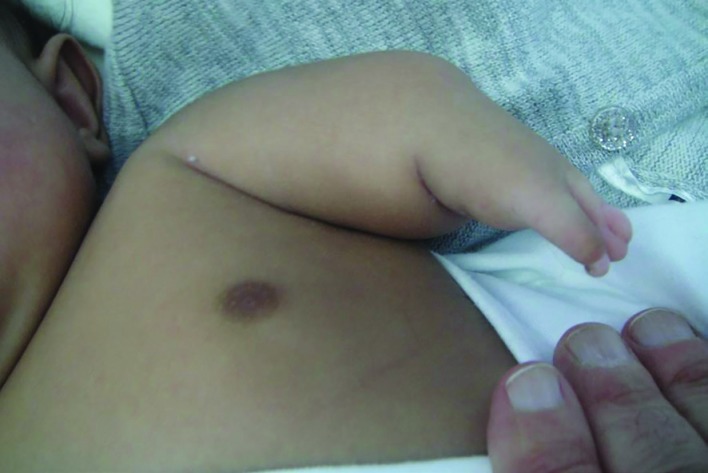
The short left upper limb. Note that the hand has only two digits

**Fig. 2:**
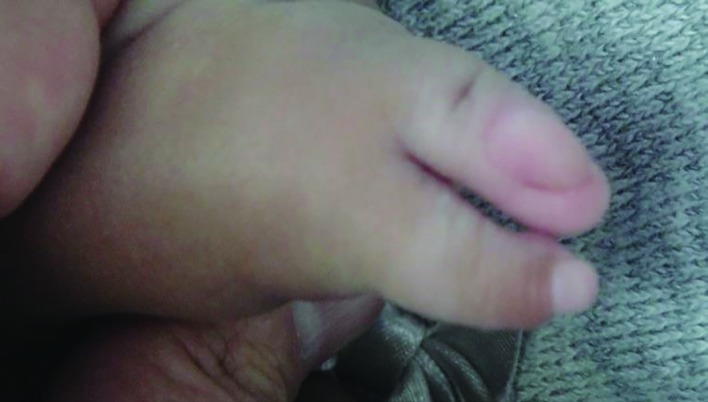
The dorsal aspect of the left hand. The ulnar digit has ventral dimelia; manifesting as absent nail along with the appearance of an ectopic pulp on the dorsal aspect of the digit

**Fig. 3:**
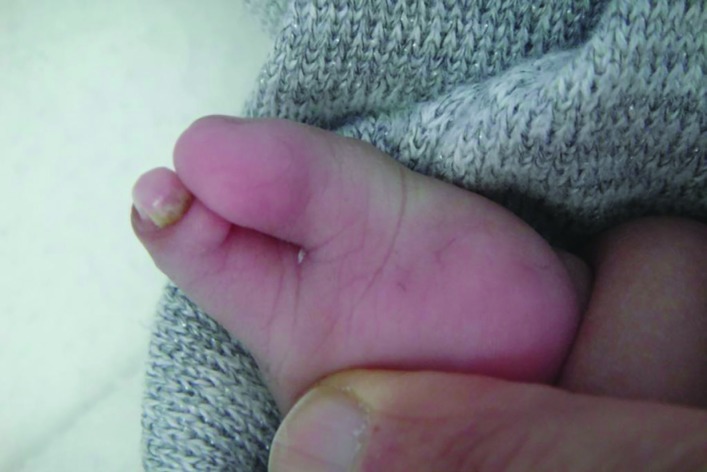
The ventral aspect of the left hand. The radial digit has dorsal dimelia; manifesting as a well-developed palmar nail and nail fold

A 9-month old Saudi female infant presented to the senior author for assessment of congenital lamb anomalies. The parents were healthy and con-consanguineous. The infant was born at full term (40 weeks of gestation) after an uneventful pregnancy and delivery. Birth weight and length were at the 30^th^ centile. Family history was negative for congenital limb anomalies. Developmental milestones were normal except for limitations related to the limb anomalies. Physical examination showed left upper limb and right lower limb anomalies. The left upper limb was short with an absent elbow joint, and the hand had two digits (Figure 1). The radial digit had dorsal dimelia; manifesting as a palmar nail. The ulnar digit had ventral dimelia; manifesting as absence of the normal dorsal nail along with the appearance of an ectopic pulp on the dorsal aspect of the digit (Figure 2 and 3). 

**Fig. 4:**
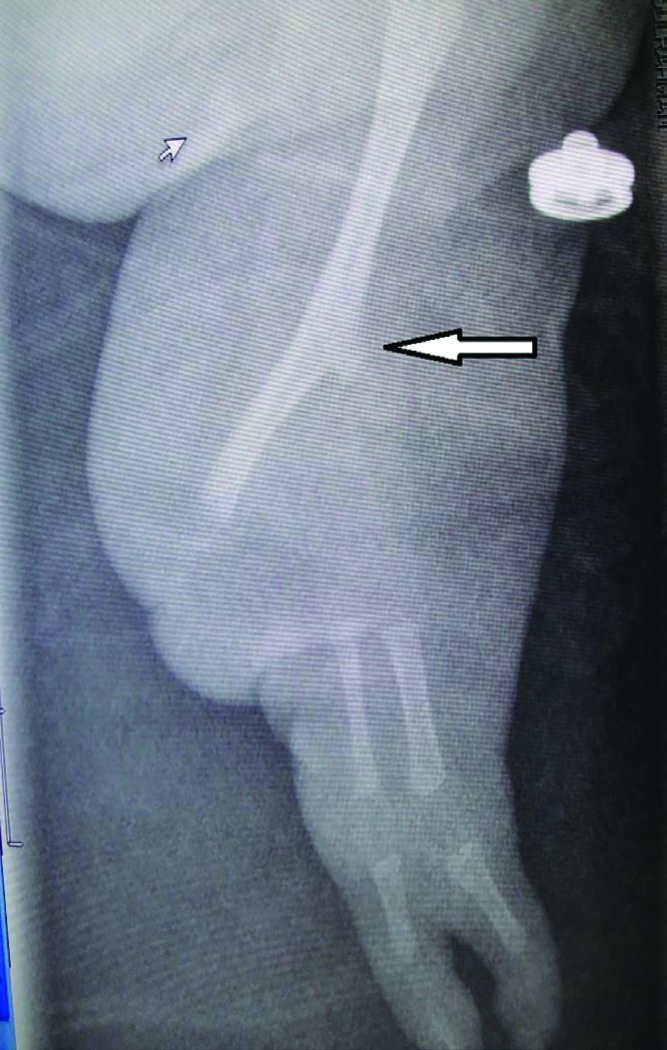
X-ray of the left upper limb showing ulnar ray deficiency. Note the radio-humeral synostosis (the area of synostosis has a prominent humeral medial epicondyle and is marked with an arrow), the absent ulna, and the two digital rays in the hand

Radiological examination of the left upper limb showed severe ulnar ray deficiency with radio-humeral synostosis and absent ulna. The hand had two metacarpals and two digital rays (Figure 4). The right lower limb was hypoplastic with three digits in the foot. The preaxial digit was a well-developed big toe. The two postaxial digits were fused (syndactyly) and hypoplastic. There was no dorsal or ventral dimelia in the affected foot. Radiological examination showed proximal focal femoral dysplasia, a short hypoplastic tibia and absent fibula. Systemic examination was unremarkable. Ultrasound examination of the brain, heart, and abdomen showed no abnormalities.

## DISCUSSION

The case we reported is unique because of the appearance of both dorsal dimelia and ventral dimelia in the same patient. The limb develops and is patterned along three axes.^8^ The proximo-distal growth is the function of fibroblast growth factors (FGF) expressed in the apical ectodermal ridge; the most important being FGF^8^ and FGF.^4^ The antero-posterior axis/patterning allows the normal development of the preaxial and postaxial structures such as the radius/thumb radially and the ulna/fingers on the ulnar side. Sonic Hedgehog (SHH) is the main controller of the antero-posterior axis. Finally, the dorso-ventral axis/patterning allows the normal development of dorsal and ventral structures of the hands and feet. Two proteins control this axis: engrailed 1 (EN-1) is expressed in the ventral ectoderm and its presence is required for the normal development of ventral structures; and the wingless protein type 7A (WNT7A) is expressed in the dorsal ectoderm and its presence is required for the normal development of dorsal structures.^8^

The genetic basis and pathogenesis of dorsal dimelia is well described in the literature. Experimentally, mice with null mutations of *En-1* develop double nails of all digits of the fore-and hind-limbs.^9^ In humans, the only syndrome in which dorsal dimelia is a constant feature of the phenotype is the distal 4ɋ deletion syndrome (deletion distal to 4ɋ 31).^10^ However, dorsal dimelia of this syndrome is always seen in one digit only (the little finger) and not in all digits as seen in experimental animals. Other features of the syndrome include micrognathia, cleft palate and cardiac anomalies. Al-Qattan^11^ noted that distal dorsal dimelia in humans may be observed with concurrent congenital hand or feet anomalies. 

In these cases, Al-Qattan^11^ noted that the distribution of dorsal dimelia usually matched the distribution of the concurrent congenital anomaly. For example, dorsal dimelia of the ulnar digits were seen with ulnar-sided anomalies such as ulnar-cleft hands, ulnar polydactyly and ulnar ray deficiency. In contrast, dorsal dimelia of the radial digits were seen with radial-sided anomalies such as radial polydactyly and radial ray deficiency.^11^ The distribution of dorsal dimelia in our case was very unusual because of the involvement of the radial digit in an ulnar ray deficiency phenotype.

The fully expressed ventral dimelia in experimental mice and humans are related to loss of function mutations in *WNT7A*.^1^ The partially expressed dimelia in humans (hypoplastic or absent nails) can be syndromal (such as the nail-patella syndrome caused by *LMX1B* mutations) or sporadic.^1^ In our case of ulnar ray deficiency, the partially expressed ventral dimelia presented as an absent nail along with the appearance of an ectopic pulp on the dorsal aspect of the ulnar digit. The pathogenesis of the combined dorsal and ventral dimelia in our case of ulnar ray deficiency is unknown. However, it is important to know that the interactions between the three axes of limb development is very complex. The ulnar ray deficiency is an error of the antero-posterior axis of development controlled by SHH.^11^

SHH is located in the posterior mesoderm and is known to be interact with other proteins in the mesoderm as well as the ectoderm. This links the antero-posterior axis to both the dorso-ventral and proximo-distal axes.^8^ This may have resulted in the combined dimelia defect seen in our patient. Finally, the clinical appearance of the left upper limb in our patient gave the impression of phocomelia with congenital absence of the entire forearm. However, a careful examination of the X-rays revealed a radio-humeral synostosis with an absent ulna. The area of synostosis is known to have a prominent humeral medial epicondyle^12^ (Figure 4). Hence, the correct diagnosis was severe ulnar ray deficiency or Bayne Type V as described by Goldfarb *et al.*^12^

## CONFLICT OF INTEREST

The authors declare no conflict of interest.
